# Erythema nodosum as first clinical sign of acute Borrelia burgdorferi infection

**DOI:** 10.1016/j.bjid.2024.103877

**Published:** 2024-09-28

**Authors:** Simona Kordeva, Lyudmil Ivanov, Valentina Broshtilova, Georgi Tchernev

**Affiliations:** a Onkoderma- Clinic for Dermatology, Venereology and Dermatologic Surgery, Bulgaria; b Department of Dermatology and Venereology, Medical Institute of Ministry of Interior, Bulgaria; c Department of Dermatology and Venereology, Military Medical Academy, Bulgaria

**Keywords:** Borrelia burgdorferi, Lyme borreliosis, Erythema nodosum, Pathognomonic symptom

## Abstract

Lyme borreliosis is a frequently encountered tick-borne infection worldwide, caused by a spirochete from the Borrelia burgdorferi genoscpecies. In most cases, the initial sign of Lyme disease is the pathognomonic symptom – erythema migrans rash appearing at the site of the thick bite. Оther described cutaneous manifestations besides erythema migrans ‒ such as erythema nodosum (an acute nodular septal panniculitis), papular urticaria, granuloma annulare, psoriatic changes, lichen striatus et atrophicans, Henoch-Schönlein purpura, and morphea ‒ could potentially present as an initial/first sign of acute Borrelia burgdorferi infection. Serological testing for Lyme disease is only reliable after the initial stages of the disease. Additional PCR or serological examinations such as ELISA, immunoblot, indirect immunofluorescence examination could be performed. The diverse cutaneous manifestations of Lyme disease can lead to delays or ineffectiveness in treatment, as these symptoms may not be promptly identified as signs of the infection. Therefore, a comprehensive evaluation of the three key aspects – clinical findings, serology, and histology – is essential and should be considered collectively. We present a 78-year-old female with an acute form of Borrelia infection following a thick bite, manifesting as erythema nodosum on the lower extremities. Serology confirmed the presence of Borrelia infection, and the histological findings were indicative of erythema nodosum. The patient initially received anti-inflammatory and antibiotic medications. Reverse development of the nodules was observed after therapy with ceftriaxone, methylprednisolone, esomeprazole, and local dressings with povidone-iodine. For outpatient care, her regimen consisted of systemic reduction of the corticosteroid therapy, esomeprazole, and doxycycline. Due to the potential triggering of erythema nodosum by valsartan, it was recommended switching to an alternative medication. The rarity of erythema nodosum as an initial or first sign of acute Borrelia infection is being discussed.

## Introduction

Lyme borreliosis, commonly referred to as Lyme disease, is a common tick-borne infection caused by the bacterial spirochete from the Borrelia burgdorferi sensu lato complex.^1^ It is transmitted through the bite of ticks belonging to the Ixodes genus, most commonly Ixodes scapularis (deer tick).^1^ The disease progresses through three stages: early localized, early disseminated, and late, each exhibiting distinct characteristics.^1^

In 70–80 % of cases, the initial sign of Lyme disease is the pathognomonic symptom-erythema migrans rash at the site of the thick bite, which is an expanding, erythematous cutaneous lesion measuring 5 cm in diameter or larger.^1^ The rash typically appears 1‒2 weeks after the initial thick bite, presenting as a homogenous erythema or a target-like lesion.^1^ Untreated, the disease can advance to more severe stage, including symptoms like arthritis, neurological issues, or cardiologic conditions.^1^

During the initial weeks of Borrelia infection, serological testing is unsensitive, and patients with erythema migrans or a history of a thick bite should begin treatment promptly.^1^

The patient's age and the stage of the disease are important in determining the appropriate treatment plan.^1^ Different antibiotics are available for treatment: doxycycline is typically preferred for most patients above 8 years old with early, localized disease, excluding children and pregnant women; cefrtiaxone is recommended as an alternative or in case of pregnancy; and amoxicillin and cefuroxime are the preferred choices for patients under 8 years old.^1^

Erythema nodosum is a frequent acute nodular septal panniculitis, marked by sudden appearance of painful, deep nodules or plaques, primarily found on the lower extremites.^2^ Women are more affected compared to the male population.^2^ It occurs commonly as an acute or recurrent type IV delayed hypersensitivity response to different antigens, infectious or noninfectious.^2^ Sarcoidosis ‒ Löfgren syndrome, characterized by a triad of erythema nodosum, acute arthritis, and hilar lymphadenopathy, is classified under the miscellaneous noninfectious causes of erythema nodosum.^2^ Further tests are required to differentiate the origin of the erythema nodosum.

We conducted a comprehensive search and gathered all available articles published in PubMed regarding Lyme disease or acute Borrelia infection presenting clinically and histologically as erythema nodosum. Our primary focus will be on this rare coexistence and discuss the potential link between them while reviewing the available literature.

## Case report

A 78-year-old female presented to the dermatology department with primary complaints of multiple erythematous-edematous lesions on the right thigh following a tick bite. She also reported pain in both legs and lower back for several days, along with swelling in her lower legs.

According to the anamnesis, the bite sites initially appeared red with a “dot” in the center (clinical suspicion for erythema migrans), which quickly developed into painful nodules. These nodules were particularly sensitive to palpation, but also caused spontaneous pain. She visited her general practitioner, who prescribed dexamethasone i.m. for three days and antihistamine tablets. The patient visited another general practitioner who prescribed cetirizine dihydrochloride 10 mg one tablet a day. Before her hospitalization, following another consultation, the patient was also prescribed amoxicillin/clavulanic acid 875 mg/125 mg twice daily for several days, bilastine 20 mg once daily, and daily bandages with povidone-iodine.

Her medical history includes arterial hypertension, which the patient manages with amlodipine 5 mg administrated once daily in the morning, and valsartan 160 mg once daily at night.

The patient presented for a physical evaluation of the lesions and further therapeutic approach to be established.

**Fig. 1:**
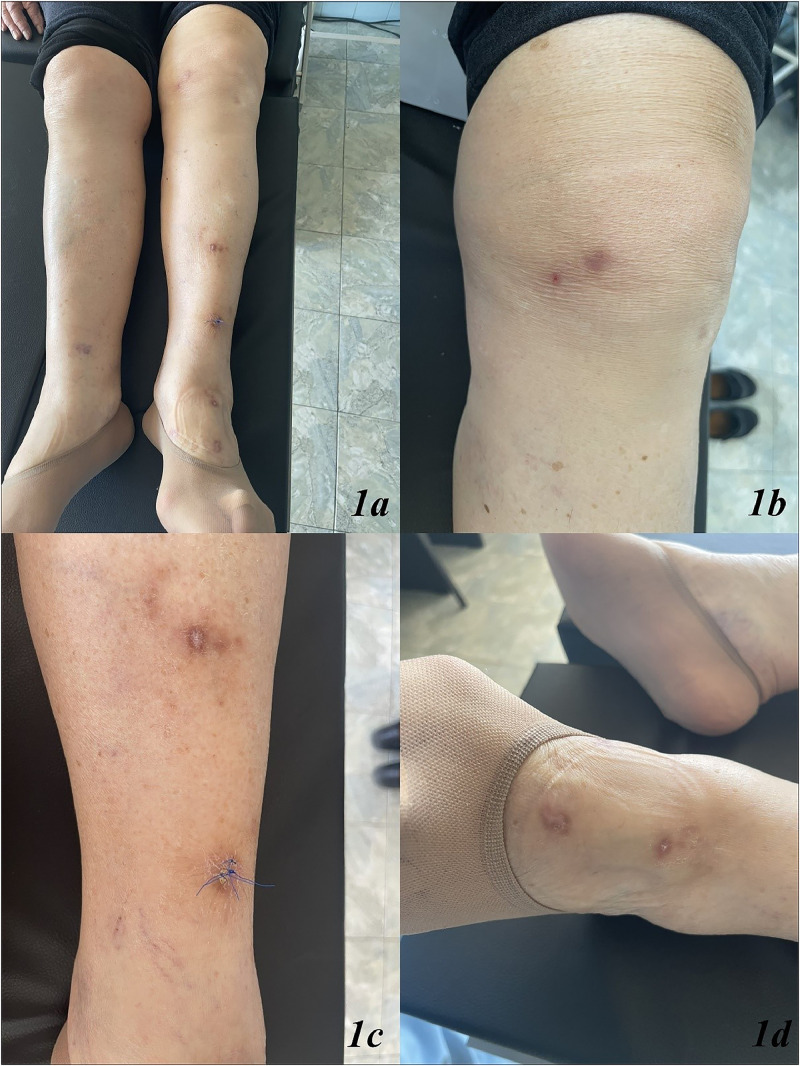
(a‒d) Multiple erythematous-edematous lesions with crusts located on the left and right lower extremities, pretibial area (a‒d). The punch biopsy was conducted from an erythema nodosum lesion located in the lower pretibial area of the left lower extremities (c).

The dermatological examination revealed multiple erythematous-edematous lesions with crusts located on the left and right lower extremities, pretibial area (Fig. 1a‒d). Enlarged lymph nodes were not palpable.

Due to clinical suspicion of erythema nodosum, laboratory tests were assigned which resulted in: ANA antibodies 1:100, ANA panel – normal; serology for Borrelia Burgdorferi – IgM – 2.05 (a positive result; referent values above 1.1 is considered positive), IgG – 0.56 (a negative result; referent values under 0.9 is considered negative). The serology for *B. Burgdorferi* was conducted using Enzyme-Linked Immunosorbent Assay (ELISA) test.

**Fig. 2:**
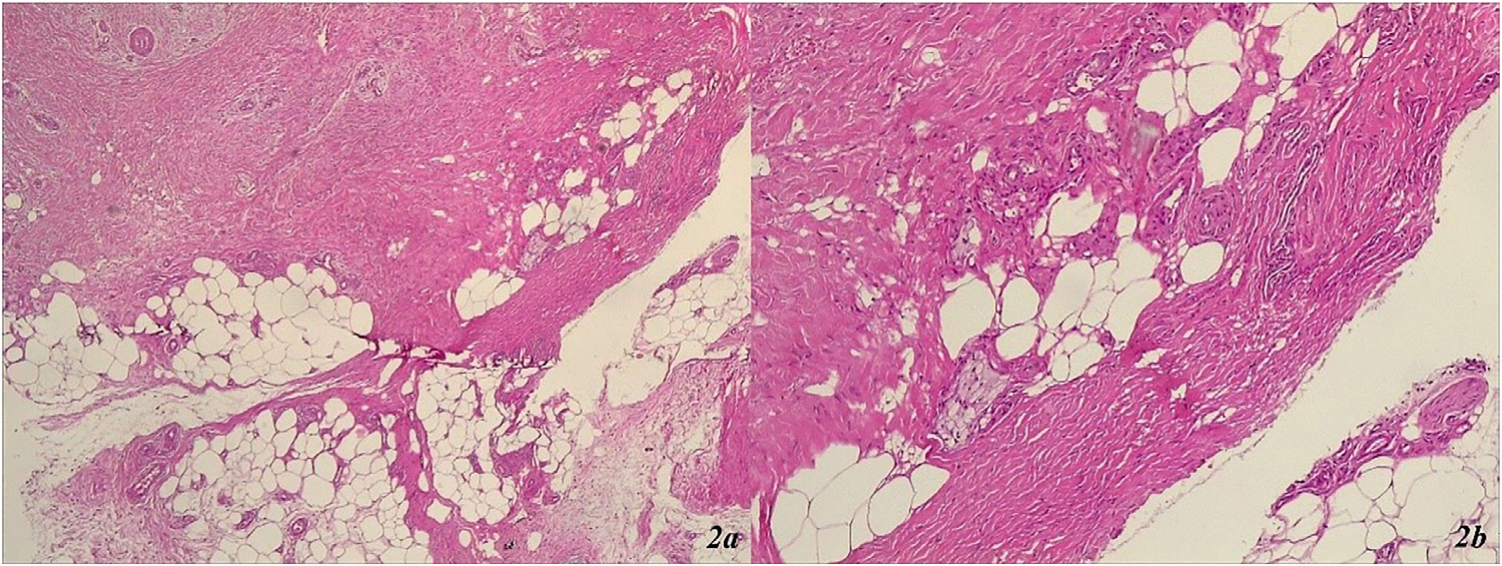
(a and b) Erythema nodosum: marked orthohyperkeratosis, irregular acanthosis, significant fibrosis throughout the dermis, septal panniculitis with the formation of dense collagen sheaths extending deep into the hypodermis. (a) Septal panniculitis × 40 × HE ‒ septal panniculitis with small foci of inflammatory cells extending into the adjacent fat lobules. (b) Septal panniculitis × 100 × HE ‒ fibrotic collagen bundles, forming thick septal compartment, extending into the adjacent lobular fat with small groups of lymphocytes and a few histiocytes.

A punch biopsy was conducted from one of the lesions in the left lower leg area (Fig. 1c), revealing marked orthohyperkeratosis, irregular acanthosis, significant fibrosis throughout the dermis, septal panniculitis with the formation of dense collagen sheaths extending deep into the hypodermis (Fig. 2a and b). The histological findings were consistent with erythema nodosum (Fig. 2a and b).

A chest X-Ray was taken to rule out Löfgren's syndrome within sarcoidosis. Due to the pain and numbness in the lower limbs, a consultation with a neurologist was scheduled, and a CT scan of the lumbar region was performed. To rule out macroangiopathy, a consultation with a vascular surgeon was appointed, who added procyanidolic oligomers 150 mg administrated twice daily to the therapy.

Treatment was initiated with ceftriaxone sodium 2 g once daily and methylprednisolone 20 mg i.v. once daily, along with esomeprazole 40 mg twice daily and local dressings with povidone-iodine. Reverse development of the nodules was observed.

For outpatient care, the following regimen was recommended: reduction of the systemic corticosteroid therapy, esomeprazole 40 mg twice daily for one month, and doxycycline 100 mg twice daily for 21 days. Due to the potential triggering of erythema nodosum by valsartan, it was recommended to switch to another medication after consulting with a cardiologist.

## Discussion

In endemic regions, medical practitioners can face significant challenges when dealing with insect bites capable of transmitting different types of diseases. The occurrence of two major-borne zoonoses in Europe, tick-borne encephalitis and Lyme borreliosis, is rising due to changes in human behavior and climate change.^3^ For the purposes of this article, the focus will be on Lyme borreliosis.

Bulgaria, located in south-eastern Europe, has limited information on Lyme borreliosis. Our review of the available medical literature indicates that the prevalence of the disease is relatively low in Bulgaria compared to other countries, making it a rare disease in this region.^4^, ^5^, ^6^ Chronologically, we will evaluate the data from the most available literature to determinate whether the low incidence rate is accurate or if it may be attributed to factors such as underreporting, misdiagnosis, false negative results, or asymptomatic infections.

Christova et al.^4^ analyzed the clinical and epidemiological data of 1257 patients diagnosed between 1999 and 2002. When comparing different age groups, the highest incidence was observed in children aged 5‒9 years, followed by adults aged 45‒49 years, 50‒54 years, and children aged 10‒14 years.^4^ The majority of patients (68 %) were either more active in or resided in rural areas.^4^ According to the study, the most prevalent clinical sign was erythema migrans, noted in 868 patients (69.1 %), followed by neuroborreliosis in 19 % of the patients, Lyme arthritis in 8 %, and heart and ocular manifestations in 1.1 % and 0.9 % of the patients, respectively.^4^ Borrelial lymphocytoma and acrodermatitis chronica atrophicans were observed in 0.3 % of the patients, while 2.1 % of patients had multiple organ involvement.^4^

Ermenlieva et al.^5^ reported that the mean annual incidence of Lyme borreliosis in Bulgaria for the period 2009‒2018 was 6.9 cases per 100,000 population, with a range between 4.1 and 11.6 cases per year. Lyme disease was found to vary significantly at the regional level, with 10-year average rates ranging from 0.3 cases per 100,000 in the Kardzhali region to 30.9 cases per 100,000 in the Gabrovo region.^5^ The northern regions of the country were identified as the most susceptible, exhibiting the highest incidence rates of the disease.^5^

Ngoc et al.^6^ stated that the annual incidence rate was 3.64 per 100,000 people over the past five years.^6^ In their study, 1892 serum samples collected in December 2023 were analyzed for specific IgG antibodies using two-tier testing protocol, which involved both ELISA and immunoblot methods. The overall seroprevalence rate was estimated at 5.4 %, with significant increases observed based on age (peaking at 8.4 % in individuals over 65), sex (8.4 % in males compared to 3.3 % in females) and residence (10.2 % in rural areas compared to 4.4 % in urban areas).^6^ Seroprevalence ranged from 0.0 % to 20.0 %, with higher rates reported in northern provinces, including Gabrovo (18.9 %) and Targovishte (20.0 %).^6^

Аlthough Lyme disease is less prevalent in Bulgaria compared to endemic regions in other parts of the world, the country has reported a relatively lower numbers of cases per 100,000 individuals. Analyzing data from the three periods covered by the above-mentioned articles ‒ 1999‒2022, 2009‒2018, and 2023 indicates a decline in incidence rates over the years. This decrease may be attributed to correct and fast diagnosis, greater public awareness, and routine medical check-ups. It is also evident that rural areas, particularly in the northern provinces of Bulgaria, present the highest risk for Borreliosis infection. After reviewing the presented papers, erythema migrans was identified as the most common clinical manifestation of Lyme disease. Less frequently reported clinical signs include Borrelial lymphocytoma and acrodermatitis chronic atrophicans. Unusual signs, such as erythema nodosum, were not mentioned in these studies.

An article by Sarah E. Randolph^3^ described the term infection prevalence as being determined by the complex interaction of diverse genetic strains of Borrelia burgdorferi with a broad spectrum of vertebrate host species, including both mammalian and avian, within an abiotic environment. Many of the host species for Ixodes ricinus vary in their competence to transmit different genospecies of Borrelia burgdorferi to the tick population.^3^ For instance, in Western Europe, rodents transmit *B. afzelii* and *B. burgdorferi* s.s., while birds transmit *B. garinii* and *B. valaisiana*.^3^ Analyzing host-tick interactions enables us to identify infection patterns and evaluate the risk of human infection.^3^

Ixodes tick-borne borrelioses refer to a group of transmissible infections caused by the spirochete Borrelia burgdorferi.^7^ These infections are characterized by chronic, progressive course, multiple organ dysfunctions, facial rashes, urticaria, and transient pointed and minor eruptions.^7^ Pathognomonic marker of the acute disease stage is the appearance of erythema migrans at the site of the thick bite.^7^ Based on the patient's medical history, the red bite sites with a central “dot” are highly indicative of erythema migrans lesions. These lesions subsequently faded following the initiation of the anti-inflammatory drug dexamethasone. A study performed by Ramesh et al.^8^ reported a significant reduction in the levels of several immune mediators induced by Borrelia burgdorferi in culture supernatants of fibroblast culture explants, microglia, astrocytes, and oligodendrocytes. Additionally, dexametasone was found to have a protective effect against Borrelia burgdorferi-induced neuronal and oligodendrocyte apoptosis in rhesus fibroblast culture explants.^8^

In a retrospective-prospective clinical study by Krkic-Dautovic et al.,^9^ treatment was administered to 509 (30.8 %) patients exhibiting symptoms of borreliosis. Skin changes accompanied by chronic symptoms affecting multiple organs occurred in 67.7 % of cases.^9^ These manifestations included pink/white borreliosis stretch marks, psoriatic changes, scleroderma circumscripta-morphae, granuloma anulare, lichen striatus et atrophicans, and erythema nodosum.^9^ 32.3 % of patient did not exhibit any skin changes.^9^ The study suggests that European borreliosis can manifest in various ways through skin changes, suggesting that erythema migrans alone does not define the disease.^9^ Erythema nodosum was found to be a rare occurrence as a skin change in patients with chronic relapsing symptoms, appearing in just 2.50 % of all cases studied, making it a relatively rare clinical finding.^9^

In another study, other cutaneous lesions besides erythema migrans, such as erythema nodosum, papular urticaria, granuloma annulare, Henoch-Schönlein purpura, and morphea, were found in patients with positive serological tests for Borrelia burgdorferi infection.^10^ Among the patients with erythema nodosum, 2 out of 9 tested positive for the spirochete.^10^ A distinctive cutaneous reaction pattern indicative of infection by reactive arthropathy-associated pathogens was suggested by Magro et al.^11^ The majority of the 16 patients included in the study developed skin lesions clinically similar to erythema multiforme, Sweet's syndrome, and/or erythema nodosum.^11^ Among the pathogens discussed in the article, Borrelia burgdorferi was also identified.^11^

These studies suggest that the above-mentioned cutaneous manifestations, especially erythema nodosum, occurring after Borrelia burgdorferi infection, though rare, could potentially serve as markers or at least be indicative of the disease in the diagnostic process.

Panniculitis as a diagnose itself is relatively rare.^12^ The smaller subset of lymphocyte-predominant panniculitis can be challenging to every pathologist due to its resemblance to T-cell lymphoma involving the subcutaneous tissue, thereby mimicking inflammatory causes of panniculitis.^12^ Erythema nodosum is a septal-predominant pattern panniculitis,^12^ characterized by bilateral and symmetric, tender erythematous nodules and plaques, measuring 1‒6 cm in diameter, mainly located in the lower extremites on the pretibial areas.^13^ While erythema nodosum is commonly categorized as a type IV delayed hypersensitivity reaction to antigenic stimuli, some authors suggest that the condition could be the result of formation and deposition of immune complexes in the venules of the septae of the subcutaneous fat.^13^^,^^14^

An acute presentation of sarcoidosis, known as Löfgren syndrome, typically includes the triad erythema nodosum, bilateral hilar lymphadenopathy and fever.^13^ Patients can also experience periarticular ankle inflammation and develop papular lesions on the knees and elbows.^13^^,^^15^ The absence of these symptoms in the patient, along with the results of the additional chest X-Ray, resulted in the ruling out of Löfgren syndrome as a diagnosis.

A tick-borne zoonosis not associated with the Ixodes ricinus complex, described by colleagues, is the Baggio-Yoshinari Syndrome (BYS), a Brazilian human borreliosis.^16^ In the article, chronic lymphomonocytic meningoencephalitis, oligoarthritis, and erythema nodosum are presented as manifestations of BYS.^16^ The causative agent, Borrelia burgdorferi sensu lato microorganisms, can mimic Lyme disease on both clinically and in laboratory evaluations.^16^ However, it can be differentiated by its prolonged clinical course, recurrent episodes, and autoimmune dysfunction.^16^

Borrelia burgdorferi antibodies can be detected using indirect immunofluorescent test.^17^ The study categorized 124 subjects into groups based on the role of Borrelia burgdorferi antibodies in patients with scleroderma circumscripta, lichen sclerosus et atrophicus, erythema nodosum, granuloma annulare, erythema annulare and chronic urticaria.^17^ These were compared to a negative control group of 131 individuals in which Borrelia burgdorferi was not considered the etiologic cause of disease, and positive group of 55 patients with lyme borreliosis.^17^ The results concluded that the negative control group had a positive titer in 44 cases (33.6 %; *n* = 131) and the positive titer was found in 9 probands within the erythema nodosum group (64.3 %, *n* = 14).^17^ The article suggests that Borrelia burgdorferi may play a role in the etiology of diseases like erythema nodosum in individuals with elevated levels of antibodies.^17^

Some authors, however, have suggested that the presence of Borrelia antibodies even in the presence of fever, joint pain and erythema, does not conclusively indicate to the diagnosis of Lyme disease.^18^ In these cases, serological examinations such as ELISA, immunoblots, two-tiered testing algorithms involving ELISA followed by immunoblots, specific antibody index measurements (calculated using the antibody titers in both serum and cerebrospinal fluid), and indirect immunofluorescence examination, can be used in diagnosing the disease.^19^

The first-line treatment for Lyme disease in nonpregnant patients aged 9 years and older is doxycycline 100 mg orally, twice daily.^20^ For patients younger than 9 years, the recommended treatment is or amoxicillin 50 mg kg^-1^ per day administered.^20^ The second-line treatment for adults with Lyme disease is amoxicillin 500 mg administered orally.^20^ Patients allergic to penicillin or cannot take tetracyclines (for example pregnant women), can be treated with cefuroxime axetil or erythromycin, with dosage regimens adjusted accordingly.^20^ Depending on the stage of the infection, therapy regimens vary: from 14 days for localized skin infections, to 30‒60 days for Lyme-associated arthritis.^20^ Acrodermatitis chronic atrophicans, commonly seen in Europe, typically requires treatment duration of 30 days.^20^ Patients with neuroborreliosis or cardiovascular manifestations require treatment with intravenous ceftriaxone, cefotaxime, or penicillin G, each for a minimum of 4 weeks.^20^

## Conclusion

While erythema migrans is widely acknowledged as the pathognomonic sign of Lyme disease, it is crucial not to overlook other cutaneous manifestations. Despite its rarity, erythema nodosum, can serve as a valuable diagnostic marker when it manifests.

After conducting a thorough review of the available literature on PubMed, we identified 10 articles linking erythema nodosum as a cutaneous manifestation to a Borrelia burgdorferi infection. This, along with the presented case report, further supports our hypothesis that erythema nodosum, when observed, could be regarded as a first/initial sign of Borrelia infection.

## Informed consent

Informed consent was obtained from all individuals included in this study.

The patients in this manuscript have given written informed consent to publication of their case details.

The authors declare that this article is intended for a case report article.

## Research funding

None declared.

## Authors’ contributions

All authors have accepted responsibility for the entire content of this manuscript and approved its submission.

## Conflicts of interest

The authors declare no conflicts of interest.
